# Limitations of Keratoplasty in China: A Survey Analysis

**DOI:** 10.1371/journal.pone.0132268

**Published:** 2015-07-10

**Authors:** Jiaxu Hong, Weiyun Shi, Zuguo Liu, Roberto Pineda, Xinhan Cui, Xinghuai Sun, Jianjiang Xu

**Affiliations:** 1 Department of Ophthalmology and Visual Science, Eye, and ENT Hospital, Shanghai Medical College, Fudan University, Shanghai, China; 2 Shandong Eye Hospital, Shandong Eye Institute, Qingdao, China; 3 Eye Institute of Xiamen University, Fujian, China; 4 Massachusetts Eye and Ear Infirmary, Harvard Medical School, Boston, Massachusetts, United States of America; Save Sight Institute, AUSTRALIA

## Abstract

**Purpose:**

Each year, over 8,000 corneal transplantation surgeries are performed in China. Unlike developed countries, which have established standard requirements for operative experience for corneal specialists, little information exists on surgical training for keratoplasty in China. The aim of this study was to assess the keratoplasty experience of Chinese corneal specialists and to characterize their surgical patterns.

**Methods:**

One hundred and twenty-one corneal specialists in 16 provinces (65 cities) in China were invited to complete an anonymous survey at the 2014 Chinese Corneal Society annual meeting, which consisted of questions with single or multiple-choice answers. Demographics, the number and type of keratoplasties performed, and the perceived limiting factors for performing keratoplasties were analyzed.

**Results:**

An overwhelming 89% response rate was achieved. Of the 108 respondents, 76% worked in tertiary centers, and only 23% held a medical doctorate degree. Furthermore, 69% of the participants had received corneal fellowship training of less than one year. Only 71% were capable of keratoplasties. Among those doing keratoplasty, 68% performed less than 50 keratoplasties each year. Of the same group of keratoplasty surgeons, 88% of corneal specialists capable of keratoplasties had performed penetrating keratoplasties, 87% had performed lamellar keratoplasties, 12% had performed deep anterior lamellar keratoplasties, and 5% had performed Descemet’s stripping endothelial keratoplasties. When questioned on the reasons for the low number of keratoplasties performed in China, the respondents deemed the following factors most important: lack of surgical training (71%), a shortage of donor supply (52%), and a lack of curricula (42%). A multivariate logistic regression analysis showed that corneal transplantation capabilities are significantly associated with responders’ education levels and training time.

**Conclusion:**

Keratoplasty surgery experience is suboptimal for Chinese corneal specialists. Penetrating and lamellar keratoplasties are the preferred surgical patterns. Our findings raise concerns about the adequacy of keratoplasty training in China.

## Introduction

The Maoist revolution in China created legions of health-care workers with limited training—called “barefoot doctors”—to provide basic medical services for millions of people. A similar situation still exists in ophthalmology. Many subspecialists’ work in Chinese eye care centers is done by the general ophthalmologists who complete a two- or three-year residency program. Corneal transplantation is a widely accepted surgical solution for blinding corneal opacity, which is a major cause of visual impairment in China. In the past ten years, the number of keratoplasties in China has increased from about 5,000 to more than 8,000.[1] Keratoplasty has advanced significantly due to the introduction of various novel surgical techniques, thereby improving postoperative visual outcomes and graft survival.[2] Over the past decade, lamellar techniques—including deep anterior lamellar keratoplasty (DALK) and Descemet's stripping endothelial keratoplasty (DSEK)—have been developed to replace penetrating keratoplasty (PKP).[3] It has been our experience that although there are huge numbers of patients waiting for keratoplasty, this surgery is performed mainly in tertiary eye centers in China, thus limiting the supply of treatment for blindness due to corneal diseases. Unlike in the United States or the United Kingdom—where the Accreditation Council for Graduate Medical Education and the Royal College of Ophthalmologists have respectively established standard requirements for operative experience for residents and fellows in keratoplasty—there are still no Chinese accreditation standards and requirements that certify whether or not a corneal specialist can perform keratoplasties. Growing evidence suggests that surgeons’ experience affects the prognosis of corneal transplantation.[3] For the first time in China, we engaged in a direct survey to determine the surgical experience of corneal specialists in keratoplasty and the factors limiting the performance of such surgeries and to characterize their surgical patterns in China.

## Methods

On behalf of the 2014 annual meeting committee of the Chinese Corneal Society (CCS), 121 corneal specialists from public hospitals and private clinics were invited to be interviewed for a survey during the course of their attendance at the meeting in Shanghai from April 24 to 27 (see Table 1 for survey questions). The aim of this study was to characterize their keratoplasty experience and surgical patterns. Furthermore, additional questions were asked to determine the limiting factors pertaining to their keratoplasty capabilities. Keratoplasties were classified as PKP, traditional lamellar keratoplasty (LKP, including central and peripheral patch grafts), DALK, or DSEK. The respondents answered the questions without providing any personal information, and the data was thus collected anonymously. The study was approved by the institutional review board and the Medical Ethics Committee of the Shanghai Eye, Ear, Nose, and Throat Hospital, and was compliant with the prevailing Health Insurance Portability and Accountability Act regulations. The research conducted met the tenets of the Declaration of Helsinki. Written informed consent was obtained from each participant before the survey.

**Table 1:** Survey Questions Regarding Keratoplasty Practice in China.

1. What is your gender?	
2. Which kind of hospital do you work for?	
3. How long have you been a corneal specialist?	
4. What academic degrees do you have?*	
A. Bachelor’s (5-year program)	B. Master’s (7- or 8-year program)
C. Doctorate (8- or 10-year program)	
5. How long was your training time as corneal specialist?	
A. Less than six months	B. Six months to one year
C. One to two years	D. More than two years
6. Can you perform keratoplasties?	
If yes, how many keratoplasties do you perform every year?	
A. 50 or more	B. Less than 50
If yes, do you perform penetrating keratoplasty?	
A. Yes	B. No
If yes, do you perform lamellar keratoplasty?	
A. Yes	B. No
If yes, do you perform deep lamellar keratoplasty?	
A. Yes	B. No
If yes, do you perform Descemet’s stripping endothelial keratoplasty?	
A. Yes	B. No
7. What do you think are the reasons for the low keratoplasty volume in China? (Select all that apply.)	
A. Lack of curricula for keratoplasty	B. Lack of surgical training
C. Not enough donors	D. Other. Please specify _______

*Master’s (7-year program = 4 years for bachelor’s courses + 3 years for master’s courses; 8-year program = 5 years for bachelor’s courses + 3 years for master’s courses) and Doctorate (8-year program = 4 years for bachelor’s courses + 4 years for doctorate courses; 10-year program = 4 years for bachelor’s courses + 3 years for master’s courses + 3 years for doctorate courses)

A statistical analysis was performed using the statistical software package SPSS for Windows (version 17.0; SPSS, Inc., Chicago, IL). The normal distribution data are shown as mean ± standard deviation. For comparison purposes, differences in the mean values of the parametric data of the different groups were analyzed using the Mann-Whitney U test. The percentage of parameters was analyzed with a chi-square test. All the variables were entered into a binary logistic regression analysis one at a time, and those variables with a probability of 0.2 or less for a relationship with the capability of performing keratoplasties were entered into a multivariate logistic regression analysis. All *P* values were two-sided and considered statistically significant when the values were less than 0.05.

## Results

### Response Rate and Respondents’ Demographics

One hundred and twenty-one corneal specialists from 16 provinces (65 cites) were invited to participate in the survey. The number of surveys completed was 108, providing a response rate of 89%. Thirteen subjects declined to participate for various reasons. Apart from 25 males, the balance of the respondents was female. Of the 108 total respondents, 82 (76%) worked in tertiary centers, while the other 26 (24%) worked in private clinics or general hospitals. The average practice time as a corneal specialist was 8.0±5.4 years, ranging from five to 25 years. To enter corneal specialist training programs in China, candidates must hold at minimum a medical bachelor’s degree. In the current study, 25 (23%) respondents reported holding a medical doctorate degree, 48 (44%) held a medical master’s degree, and 35 (32%) had a medical bachelor’s degree. In terms of corneal training, 39 (36%) reported participating in corneal fellowship training for less than six months, 35 (32%) reported receiving training for between six months and one year, 13 (12%) reported between one and two years of training, and 21 (20%) reported more than two years of training.

### Surgical Experience

Of the 108 respondents, 77 (71%) reported that they had performed keratoplasties, whereas 31 (29%) were not capable of this surgery, as shown in Table 2. A chi-square analysis revealed that there were no statistically significant differences in keratoplasty capability between the male and female subjects (*P* = 0.066) or between those practicing in different workplaces (*P* = 0.054). Those respondents with longer practice times (*P* = 0.036), higher medical degrees (*P* = 0.006), and more training time (*P* = 0.013) tended to be capable of performing keratoplasties. However, the multivariate logistic regression analysis revealed that only academic degrees (*P* = 0.024) and training time (*P* = 0.039) were limiting factors associated with keratoplasty capability (Table 3).

**Table 2:** Keratoplasty Experience among Chinese Corneal Specialists.

Parameters	Capable of Keratoplasty	Not Capable of Keratoplasty	*P* value*
**Respondents (n)**	77	31	n/a
**Gender**			0.066
Male	29	6	
Female	48	25	
**Workplace**			0.054
Tertiary center	63	20	
Non-tertiary center	14	11	
**Practice time (years)**	8.7 ± 6.1	6.1 ± 5.3	0.036
**Academic degree**			0.006
Doctorate	21	4	
Master’s	38	10	
Bachelor’s	18	17	
**Training time**			0.013
Less than six months	26	13	
Six months to one year	20	15	
One year to two years	11	2	
More than two years	20	1	

n/a = not applicable

* A chi-square test and a Mann–Whitney U test were performed between the groups.

**Table 3:** Multivariate Logistic Regression Analysis of Potential Limiting Factors Associated with the Capability of Chinese Corneal Specialists to Perform Keratoplasties.

Parameter	*P* Value
**Gender**	0.244
**Workplace**	0.542
**Practice time**	0.059
**Academic degree**	0.024
**Training time**	0.039

Among those performing keratoplasties, 52 (68%) performed less than 50 keratoplasties each year, and only 25 (32%) reported performing more than 50 keratoplasties each year. In this group, we found that surgical volumes did not correlate to gender (*P* = 0.426), workplaces types (*P* = 0.731), practice time (*P* = 0.206), medical degrees (*P* = 0.769), or training time (*P* = 0.166).

### Keratoplasty Pattern

As shown in Table 4, of the 77 specialists who had performed keratoplasties, 68 (88%) had performed PKP, 67 (87%) had performed LKP, 9 (12%) had performed DALK, and 4 (5%) had performed DSEK. Specialists who were performing ≥50 keratoplasties per year were associated with higher percentages of DALK and DSEK performance (Table 4).

**Table 4:** Keratoplasty Patterns among Chinese Corneal Specialists.

0050	≥ 50 Keratoplasties Each Year	< 50 Keratoplasties Each Year	*P* value*
**Respondents (n)**	25	52	n/a
**Penetrating keratoplasty**			0.485
Yes	23	45	
No	2	7	
**Lamellar keratoplasty**			0.586
Yes	21	46	
No	4	6	
**Deep anterior lamellar keratoplasty**			< 0.001
Yes	8	1	
No	17	51	
**Descemet’s stripping endothelial keratoplasty**			0.003
Yes	4	0	
No	21	52	

n/a = not applicable

* A chi-square test was performed between the groups

### Reasons for Low Keratoplasty Volumes in China

In terms of the reasons for the low keratoplasty volumes in China, 77 (71%) respondents felt that a lack of surgical training was an important limiting factor, followed by a shortage of donor supply (56 respondents, 52%), and a lack of curricula (45 respondents, 42%). Whether respondents were capable of performing keratoplasties or not did not affect their answers (all *P* > 0.05, Table 5). It should be noted that multiple responses were allowed, so the percentages add up to more than 100%.

**Table 5:** Reasons for Low Keratoplasty Volumes in China.

Parameters	Capable of Keratoplasty ()	Not Capable of Keratoplasty	*P* value*
**Respondents (n)**	77	31	n/a
**Lack of surgical training**			0.604
Yes	56	21	
No	21	10	
**Shortage of donor supply**			0.975
Yes	40	16	
No	37	15	
**Lack of curricula**			0.640
Yes	31	14	
No	46	17	

* A chi-square test was performed between the groups.

n/a = not applicable

## Discussion

During the current period of complex and dramatic change in the Chinese healthcare environment, the practices of corneal specialists are facing many challenges, including large numbers of patients with corneal blindness, a lack of corneal donors, and limited medical funding. In addition, corneal fellowship training programs have difficulty providing a solid knowledge base and the appropriate surgical skills to trainees; indeed, fellowship programs are still not common in China.

In general, around three-quarters of the corneal specialists who participated in this survey came from tertiary centers. Even so, only about a third had participated in corneal fellowship training of more than one year, and less than a quarter had a medical doctorate degree. Seventy-one percent of respondents reported being capable of performing keratoplasties. PKP and traditional LKP remained the main surgical choices for most Chinese corneal specialists. Very few cornea surgeons were capable of performing the DALK and DSEK in this survey. More than half of the participants felt that training and donor tissue were limitations for the practice of keratoplasty in China, indicating the need to improve surgical training and increase the supply of corneal donors. Interestingly, we found that that lower academic degrees and shorter training time were the limiting factors associated with the specialists’ keratoplasty capabilities.

The keratoplasty experience of Chinese corneal specialists has not previously been reported. Although data is lacking, ophthalmology residency and fellowship training programs have not been systematically established in China, potentially further raising the likelihood of insufficient, or even absent, keratoplasty experience among corneal specialists. Not surprisingly, we found that 29% of the respondents had no keratoplasty surgical experience. In addition, only a minority of the respondents had performed DALK and DSEK. The respondents capable of surgeries seemed to have higher education levels and longer training times. However, these factors did not correlate with the surgical volumes undertaken by these surgeons. A possible reason is that the practice of keratoplasty in China is not only limited by low education levels of corneal specialists, but also by the lack of cornea donors. A similar lack of surgical experience and low surgical volumes have also been reported for cataract surgery among Chinese ophthalmologists.[4] Our data suggest that there is room to enhance corneal specialist education and training in China, which in turn may result in improvements in surgical capabilities and techniques and thus in service to patients.

While the majority of respondents had performed PKP and LKP in their surgical practices, only a few had DALK and DSEK experience. There is a definite learning curve for both DALK and DSEK procedures.[2, 3] Although most of the cornea surgeons in our study possessed the required skills for PKP, those working in the community had had few opportunities to perform such surgeries. Our data support the existence of a learning curve for these new techniques: corneal specialists performing ≥50 keratoplasties per year reported more DALK and DSEK surgeries than those performing less than 50 keratoplasties. We did not investigate the effect of primary corneal diseases on the surgical patterns. However, it can be assumed that patient selection varies according to the different levels of hospitals. Nevertheless, this effect seems to be limited; most of the respondents came from tertiary centers. Our findings suggest that surgical experience and volumes may limit corneal specialists’ surgical pattern choices, again emphasizing the need for corneal specialist training programs in China. For established cornea specialists who wish to learn DSEK and DALK, a coordinated effort with an eye bank and academic center is recommended.

The reasons for the low keratoplasty volumes in China are multifactorial. More than half of the respondents identified a lack of surgery training and a shortage of donor supply as the main limiting factors. These results did not differ whether respondents were capable of performing keratoplasties or not. It has been pointed out that a lack of training is a major barrier to the performance of keratoplasty.[5] A recent study revealed that most Chinese hold negative attitudes toward cadaveric organ donation, resulting in diminished donor supply.[6] In addition, Chu et al. found that the registered donors have more positive attitude towards cornea donation than control subjects do, meaning that the improvement of Chinese corneal donor registration system could be beneficial to promote the cornea donation.[7] Our findings suggest that both administrators and trainers should emphasize keratoplasty in residency and fellowship training and actively promote cadaveric organ donation.

Our study has some inherent limitations. First, all of the respondents provided self-reported data on their surgical experience and patterns, and this may have introduced ascertainment and recall biases. To address this issue, most of the answers were designed to be “yes” or “no”. Second, the surgical experience and patterns for keratoplasty in this group may be different from those of a general cohort because the survey was obtained in the CCS annual meeting, resulting in a possible selection bias with respect to the number of Chinese corneal specialists. However, as the largest professional organization of corneal specialists, the CCS provides its members with ongoing medical education and information regarding advances in corneal disease research. Although official data were unavailable, most of the Chinese corneal specialists who are capable of keratoplasties would have attended this meeting. We believe that our results therefore probably overestimate rather than underestimate corneal specialists’ experience with keratoplasty in China.

The lack of keratoplasty experience remains a significant issue for Chinese corneal specialists. In addition, their education levels and involvement in training programs need to be improved. Standards and requirements for surgical training programs need to be established and/or upgraded urgently to facilitate corneal sub-specialization certification. We hope that our work will stimulate further investigation into the quality and quantity of resident and fellow training in this surgical specialty not only in China but also in other developing countries.
